# An order parameter for synchronisation of angular velocities

**DOI:** 10.1140/epjs/s11734-025-01984-3

**Published:** 2025-11-03

**Authors:** Julian Newman, Joe Rowland Adams, Philip T. Clemson, Aneta Stefanovska

**Affiliations:** 1 Department of Mathematics and Statistics, University of Exeter, Exeter, UK; 2 Physics Department, Lancaster University, Lancaster, LA1 4BY UK; 3 Department of Mathematics and Statistics, Lancaster University, Lancaster, LA1 4BY UK; 4 Aston Digital Futures Institute, Aston University, Birmingham, B4 7ET UK

## Abstract

Order parameters have proved a vital tool for simplifying and understanding complex dynamics in a range of physical systems. However, applying these approaches to the high-dimensional time-dependent variability inherent to thermodynamically open systems—such as those found in neural networks and climate dynamics—remains a challenge. We introduce a novel order parameter based on alignment of component frequencies, in contrast to the widely used Kuramoto order parameter’s alignment of process phases. We present numerical simulations comparing this new parameter to the Kuramoto order parameter in a range of models, including a prototypical phase-oscillator model relevant to many open systems, such as neuronal dynamics. These results show that the new parameter more accurately identifies synchronisation in conditions characteristic of open systems, revealing dynamics entirely missed by established methods.

**Supplementary Information:**

The online version contains supplementary material available at https://doi.org/10.1140/epjs/s11734-025-01984-3.

## Introduction

The concept of an order parameter was introduced independently by Gorsky [1] and Bragg and Williams [2] to describe order–disorder transition of alloys. It was developed in its modern form by Landau [3] to provide a phenomenological description of phase transitions. It was extended by Haken [4] to an ‘enslaving-principle’ in which dynamics is governed by a few order parameters due to external forcing. This latter contribution—serving as a dynamical-systems-theory analogue to Prigogine’s thermodynamic theory of dissipative structures [5]—particularly helped to extend the application of order parameters to non-equilibrium behaviour in thermodynamically open systems, where external forcing is ubiquitous. One of the most prevalent order parameters in dynamical systems was proposed by Kuramoto [6, 7], within a framework of phase dynamics, to capture the degree of synchrony amongst a collection of phase oscillators. The Kuramoto order parameter has been widely used to describe the degree of synchronisation [8–12], which is a fundamental and universal phenomenon throughout the sciences, including both classical and quantum physics [13–15], and is essential to the robust functioning of, *inter alia*, biological systems [16–23]. However, most of the study of synchronisation and application of order parameters for synchronisation has been in the setting of infinite-time autonomous dynamical systems, where the intrinsic time-variability central to the functioning of biological and other thermodynamically open systems is entirely missed. In this article, we introduce a new order parameter for phase-oscillator networks based on synchronisation of angular velocities, motivated particularly by the limitations of previous approaches for studying time-dependent dynamics.

Synchronisation is classically concerned with the alignment of the frequencies of interacting rhythmic processes. Such processes can be effectively represented by oscillator networks [11], of which Kuramoto oscillators are a leading example [6, 7]. These include both autonomous (i.e. not externally forced) networks as well as deterministically non-autonomous [24–26] and stochastically forced [9, 27–29] oscillator networks. More precise concepts of synchronisation include:

(i) The synchrony of the rates of progression of the oscillators through their respective cycles, without concern for the absolute positions along the cycle across different oscillators. The strongest kind of synchronisation in this category is called *frequency synchronisation* or *phase locking* [11, 13, 30]. This is where the angular velocities of the oscillators tend towards being identical.
(ii) Those that concern the extent to which the oscillators share approximately the same positions along their cycles, as opposed to merely progressing through their respective cycles at the same rate. The strongest kind of synchronisation in this category is called *phase synchronisation* [11]. This is where the phases of the oscillators tend towards being identical.

Another important distinction when discussing synchronisation is that of finite-timescale behaviour versus long-time-asymptotic behaviour [31, 32]. For models with time-independent dynamics (autonomous models) or models whose time-dependence follows a fixed infinite-time deterministic or statistical pattern, it is often natural to analyse dynamics at the level of long-time-asymptotic behaviour, and synchronisation has usually been addressed at that level. However, for systems such as biological systems that need to be able to perpetually adapt their internal rhythms to their ever-changing environment, time-independent models are not appropriate and long-time-asymptotic analysis of dynamics is not suitable, and so synchronisation should be considered on finite timescales.

The physical phenomenon of synchronisation is most often defined qualitatively using (i) [9]. Nevertheless, for quantification of synchronisation, order parameters have been defined both in terms of alignment of frequencies and alignment of phases. The most commonly used order parameter for studying synchronisation of phase oscillators is the *Kuramoto order parameter* [6, 7], which measures alignment of phases. At each instant in time, it takes a value in the interval [0, 1], where a value of 1 corresponds precisely to the scenario that all the oscillators have exactly the same phase at that instant in time. Thus, the Kuramoto order parameter really quantifies synchrony in the sense of (ii). One natural consequence is that it will not detect synchronisation of frequencies in situations where this does not correlate to alignment of phases; this is particularly important for networks with repulsive or with *mixed attractive and repulsive* [16, 30, 33, 34] coupling, which lie at the forefront of contemporary research in oscillatory dynamics including biological oscillators. Another important point is that for autonomous networks with a critical coupling strength for frequency synchronisation, even if the Kuramoto order parameter is correlated to the coupling strength, the Kuramoto order parameter will take values below 1 when the coupling strength is slightly above the critical value [30, 35]; we will see that this then has an important consequence for nonautonomous models: Despite being an explicitly time-dependent order parameter and hence applicable to nonautonomous systems, the Kuramoto order parameter will generally fail to clearly reveal temporally intermittent synchronisation in nonautonomous systems even when those systems take a form in which the Kuramoto order parameter is correlated to the coupling strength.

For systems where long-time-asymptotic analysis is applicable, time-independent order parameters for frequency synchronisation have been developed, such as ones quantifying the proportion of oscillators taken up by a maximal frequency-synchronised collection [36, 37], and one quantifying the long-time-asymptotic alignment of phase growth in analogy to how the Kuramoto order parameter quantifies the instantaneous alignment of phases [38]. In [37], the instantaneous variance of angular velocities has been applied in the context of studying frequency synchronisation in an autonomous thermodynamically-limiting lattice of phase oscillators.

Our new order parameter is an explicitly time-dependent order parameter for quantifying synchronisation in the sense of (i), analogous to how the Kuramoto order parameter quantifies synchronisation in the sense of (ii). It is based on the instantaneous variance of angular velocities, but suitably transformed to make it an order parameter in the interval [0, 1] that is appropriately applicable not only to autonomous systems but also to nonautonomous systems with gradually time-dependent natural frequencies. It measures at each instant in time how close the network’s coupling brings the oscillators to time-localised phase locking: it gives a value of 0 when there is no coupling, and a value of 1 when the oscillators have exactly the same angular velocity. Thus, by supplementing the Kuramoto order parameter with our new order parameter, our understanding of the synchronisation that occurs in widely physically relevant models is brought closer to the physical reality of synchronisation.

The paper is organised as follows: In the next section we define the new angular frequency order parameter and make clear its relation to the Kuramoto order parameter. After that, we contrast the two order parameters in

(i) a simplest-case two-oscillator system,
(ii) more physically realistic higher-dimensional networks with autonomous and non-autonomous oscillators,
(iii) mixed attractive-repulsive coupling of multiple forms in higher-order autonomous and non-autonomous networks, representing complex systems such as neuronal dynamics.

Finally, we discuss macroscopic-phase descriptions of oscillator networks associated with each of the two parameters, which is of important relevance to network outputs and inter-network interactions.

## Derivation of the order parameter

We begin by recalling the definition of the Kuramoto order parameter. Given a network of *N* phase oscillators $\theta _1,\ldots ,\theta _N$, where at each time *t* the state $\theta _j(t)$ of the *j*-th oscillator $\theta _j$ is an angle between 0 and 2π, the *mean field* of the network is defined as

$$\begin{aligned} \mu (t) = \frac{1}{N} \sum _{j=1}^N e^{i\theta _j(t)}. \end{aligned}$$

It is a complex-valued function of time, taking values in the unit disc. The magnitude r(t):=|μ(t)| is known as the Kuramoto order parameter of the network. At any time *t*,

1 $$\begin{aligned} r(t) = 1 \quad \Longleftrightarrow \quad \theta _1(t) = \theta _2(t) = \cdots = \theta _N(t). \end{aligned}$$

When $\mu (t) \ne 0$, we can also consider the argument $\phi (t):=\textrm{arg}(\mu (t))$, called the *mean phase* of the network.

To be able to carry over the concept of the Kuramoto order parameter from its original setting of alignment of phases to the new setting of alignment of angular velocities, we first need to understand how the Kuramoto order parameter relates to the *variance* of the phases. In a linear geometry, the (population) variance of a list of numbers $x_1,\ldots ,x_N \in {\mathbb {R}}$ is the mean of the squared deviations from $\textrm{mean}(x_1,\ldots ,x_N)$. For a phase-oscillator network, regarding the phase $\theta _j(t)$ of each oscillator as a value on the unit circle, we define the variance of the phases at a given time *t* to be the mean of the squared Euclidean distances from the mean phase; that is,

$$\begin{aligned} \textrm{var}(t) = \frac{1}{N} \sum _{j=1}^N \left| e^{i\theta _j(t)} - e^{i\phi (t)} \right| ^2. \end{aligned}$$

This assumes that $\mu (t) \ne 0$, since ϕ(t) is not well-defined when μ(t)=0. When μ(t)=0, one can show (see Sec. I of the Supplementary Information for a proof) that

$$\begin{aligned} \frac{1}{N} \sum _{j=1}^N \left| e^{i\theta _j(t)} - e^{i\phi } \right| ^2 = 2 \quad \forall \, \phi \in [0,2\pi ), \end{aligned}$$

and so in this case, we simply take $\textrm{var}(t)=2$. Having defined the variance of a phase-oscillator network, one can show (see Sec. I of the Supplementary Information for a proof) that the Kuramoto order parameter *r* is related to the variance by

2 $$\begin{aligned} 1-r(t) = \frac{\textrm{var}(t)}{2} = \frac{\text {actual variance}}{\text {variance in absence of order}}. \end{aligned}$$

By “variance in absence of order,” we mean the variance when r=0, which we have said is indeed equal to 2.

We now go on to define our new order parameter. It is defined for phase-oscillator networks where:

- Each of the *N* oscillators $\theta _j$ is assumed to have an *internal frequency* $f_j(t)>0$ (which is allowed to depend gradually on time *t*), where, in the absence of coupling with the other N-1 oscillators in the network, the phase of $\theta _j$ would approximately evolve as ${\dot{\theta }}_j(t)=2\pi f_j(t)$.
- The *N* oscillators do not all have the same internal dynamics. Specifically, we will assume that at every time *t*, we do not have $f_1(t)=f_2(t)=\ldots =f_N(t)$.
- The phases $\theta _j(t)$ evolve as differentiable functions of time (as opposed to, say, being governed by a white-noise-driven stochastic differential equation), where the contributions to the angular velocity ${\dot{\theta }}_j(t)$ are the internal-dynamics component $2\pi f_j(t)$ and the coupling to the other oscillators in the network.

From a practical point of view: The “internal frequency” models a natural frequency for the mechanisms responsible for producing oscillations. For example, a spring-mass oscillator with slowly varying stiffness $k_j(t)$ and slowly varying mass $m_j(t)$ would have an internal frequency $f_j(t)=\frac{1}{2\pi }\sqrt{\frac{k_j(t)}{m_j(t)}}$; and more sophisticated real-world oscillators can also often be understood as analogously having an internal frequency arising from their mechanism of oscillations. However, if there is a relatively sharp change in internal frequency, then our order parameter cannot meaningfully be applied during the time that the sharp change is taking place. The second of the three points above is saying, practically speaking, that the oscillators cannot all be identical or near-identical copies of each other; the different oscillators must have different internal frequencies. The third of the three points is saying that our order parameter, in its current form, cannot be applied when there is significant dynamical noise or other very-fast-timescale behaviour (compared to the internal frequencies) present in the phase component of the oscillator dynamics. Sometimes dynamical noise can significantly affect the amplitude components while still allowing the extraction of sufficiently smooth time-series for the phases of the oscillators, in which case our order parameter is still applicable. A direction of future research would be to introduce time-windowed versions of the order parameter better suited for dealing with dynamical noise. The issue of dealing with measurement noise will be addressed towards the end of this paper.

For a system fulfilling the above three points, writing $\omega _j(t)=2\pi f_j(t)$, our order parameter *R*(*t*) is defined as

3 $$\begin{aligned} R(t) = \max \!\left( 1 - \frac{\textrm{variance}({\dot{\theta }}_1(t),\ldots ,{\dot{\theta }}_N(t))}{\textrm{variance}(\omega _1(t),\ldots ,\omega _N(t))} \,, \, 0 \right) \!, \end{aligned}$$

where the ‘variances’ refer to the usual (i.e. linear-geometry) definition of variance. In terms of the analogy to equation (2), $\textrm{variance}({\dot{\theta }}_1(t),\ldots ,{\dot{\theta }}_N(t))$ represents the variance of the actual angular velocities of the network, while $\textrm{variance}(\omega _1(t),\ldots ,\omega _N(t))$ represents the variance of what the angular frequencies of the oscillators would be if their dynamics were not coupled to each other. So, at any time *t*, having the former variance equal to the latter would give a value of R(t)=0, representing an absence of coupling-induced order. If, at some time *t*, the presence of coupling causes the angular velocities to have *greater* variance than the internal angular frequencies, we still simply regard there as being no coupling-induced order; and so, rather than allowing *R*(*t*) to become negative, we simply cut it off at 0. Thus, *R*(*t*) takes values in the unit interval [0, 1], like *r*(*t*) does. In analogy to Eq. (1), we have

$$\begin{aligned} R(t) = 1 \quad \Longleftrightarrow \quad {\dot{\theta }}_1(t) = {\dot{\theta }}_2(t) = \cdots = {\dot{\theta }}_N(t). \end{aligned}$$

At each time *t*, the dependence of *R*(*t*) on $(\dot{\theta _1}(t),\ldots ,{\dot{\theta }}_N(t))$ is continuous, but is not differentiable at the boundary between R=0 and R>0. This is somewhat akin to how the dependence of the Kuramoto order parameter *r* on $(\theta _1,\ldots ,\theta _N)$ is continuous, but is not differentiable at r=0. For both order parameters, an everywhere-differentiable version of the order parameter can be obtained simply by squaring it (still giving a value in the unit interval [0, 1]).

## Illustrative examples

We now illustrate the order parameter *R*(*t*) and its comparison with the Kuramoto order parameter *r*(*t*). Our examples will be ordinary differential equations of the form

4 $$\begin{aligned} {\dot{\theta }}_j(t) = \omega _j(t) + g_j(\theta _1(t),\ldots ,\theta _N(t),t), \quad j=1,\ldots ,N. \end{aligned}$$

Phase-oscillator network models of this form can often be derived from higher-dimensional oscillator networks via *phase-reduction* [39–41]. Such a system is *autonomous* if for every *j*, the value $\omega _j(t)$ and the function $g_j(\,\cdot ,\ldots ,\,\cdot ,t)$ do not depend on *t*; otherwise, it is *non-autonomous*. The former thus represents a closed system, and the latter an open one. Just as the Kuramoto order parameter can be constructed as an observable over the $(\theta _1,\ldots ,\theta _N)$-state space by

$$\begin{aligned} r(t) =&\ {\tilde{r}}(\theta _1(t),\ldots ,\theta _N(t)) \\ {\tilde{r}}(\theta _1,\ldots ,\theta _N) :=&\ \frac{1}{N}\left| \sum _{j=1}^N e^{i\theta _j} \right| , \end{aligned}$$

so likewise our new order parameter can be constructed as a time-dependent observable over the $(\theta _1,\ldots ,\theta _N)$-state space by

5 $$\begin{aligned} R(t) =&\, {\tilde{R}}(\theta _1(t),\ldots ,\theta _N(t),t) \end{aligned}$$

6 $$\begin{aligned} {\tilde{R}}(\theta _1,\ldots ,\theta _N,t) : =&\max \!\left( 1 - \frac{\textrm{variance}\big (\omega _j(t) + g_j(\theta _1,\ldots ,\theta _N,t) : j \in \{1,\ldots ,N\}\big )}{\textrm{variance}\big (\omega _j(t) : j \in \{1,\ldots ,N\}\big )} \, , \, 0 \right) \!. \end{aligned}$$

**Fig. 1:**
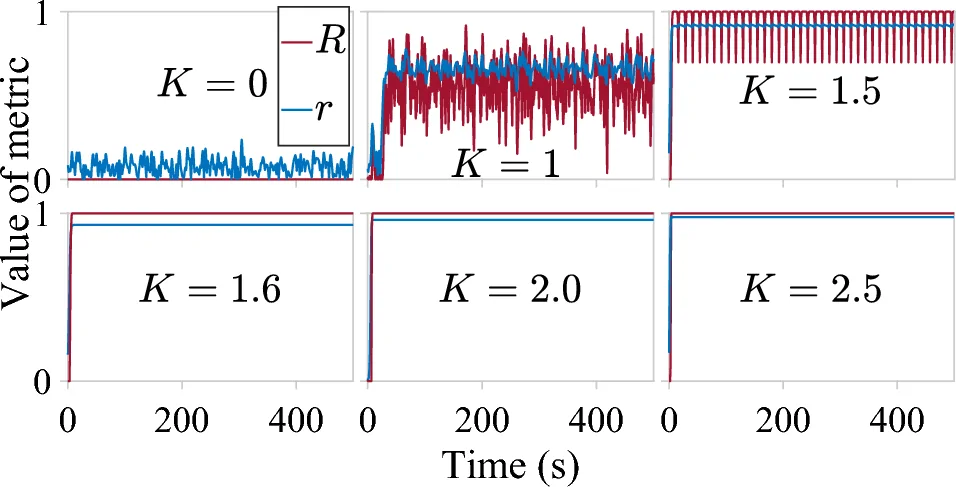
The order parameters for the autonomous Kuramoto network specified in (8) with N=100, $\mu _\omega =\pi$ rad/s and $\sigma _\omega =0.5$ rad/s, and with coupling strength *K* having the indicated values as measured in rad/s. The Kuramoto order parameter (blue line) takes a non-zero value for the case of zero coupling. It can then be seen to asymptotically converge to 1 as the coupling strength is increased. In contrast, the new order parameter (red line) starts at a value of 0 for the zero coupling case and has a value of 1 for all coupling strengths above the critical coupling for frequency synchronisation

We compute *R*(*t*) by (5)–(6) applied to the numerically obtained solution $(\theta _1(t),\ldots ,\theta _N(t))$. All numerics were performed using a Runge–Kutta 4-step numerical integration algorithm with an integration step of 0.001 s. For each system under consideration, the initial phases $\theta _j(0)$ for the simulations are randomly sampled from the uniform distribution on $[-\pi ,\pi ]$; when varying the coupling strength *K* in a given model, the same random values of $\theta _j(0)$ were used across the varying *K*.

### Two-oscillator system

We first consider the simplest example, namely an autonomous pair of coupled oscillators

7 $$\begin{aligned} {\dot{\theta }}_1(t)&= \omega _1 + \frac{K}{2}\sin (\theta _2(t)-\theta _1(t)) \nonumber \\ {\dot{\theta }}_2(t)&= \omega _2 + \frac{K}{2}\sin (\theta _1(t)-\theta _2(t)), \end{aligned}$$

with constants $\omega _2>\omega _1>0$ corresponding to the internal angular frequencies of $\theta _2$ and $\theta _1$ respectively, and a coupling strength constant $K \in {\mathbb {R}}$.

- For |*K*| very small, the new order parameter assigns near-perfect disorder (R≈0), while the Kuramoto order parameter *r* oscillates over the full range between 0 and 1.
- For $|K|>\omega _2-\omega _1$, there is a stable phase-locked solution, corresponding to ‘perfect order’ under the new order parameter (R=1). In the case of large positive *K* (attractive coupling), the Kuramoto order parameter similarly assigns near-perfect order (r≈1) as the two oscillators are approximately in phase. However, in the case of large negative *K* (repulsive [42] coupling), the Kuramoto order parameter assigns near-perfect disorder (r≈0) as the two oscillators are approximately in antiphase, despite exhibiting frequency synchronisation.

Hence, it is already evident from this simplest example how the new angular velocity order parameter aligns with the physical concept of synchronisation in cases where the Kuramoto order parameter does not. See Sec. II of the Supplementary Information for further theoretical calculation details and numerics.

**Fig. 2:**
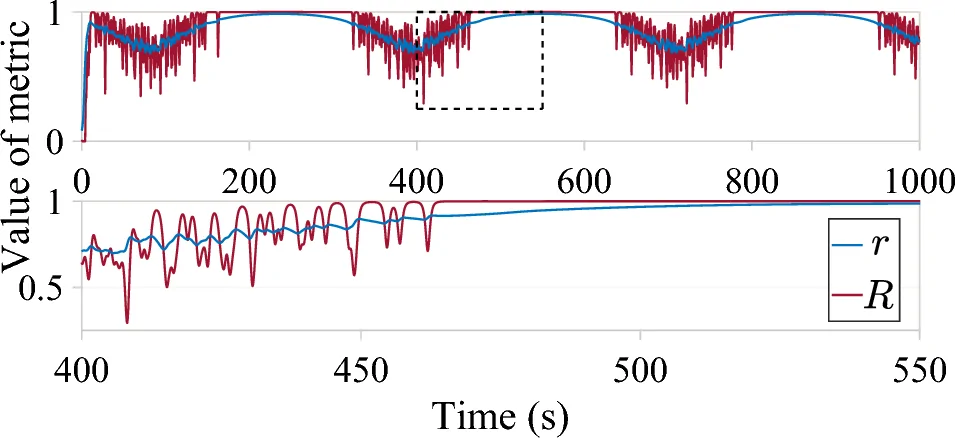
The order parameters for the non-autonomous Kuramoto network specified in (9) with N=100, $\mu _\omega =\pi$ rad/s, $\sigma _\omega =0.5$ rad/s, M=0.5, $\omega _m=0.1$ rad/s and K=1.578 rad/s. The lower plot is a zoomed-in picture of the dashed-rectangled region of the upper plot. The new order parameter (red line) reveals intermittent synchronisation in the system, staying at 1 during synchronised states but oscillating over a broader range during non-synchronised states. The Kuramoto order parameter (blue line) shows subtle alternations between smoother and rougher time-evolution, but does not clearly reveal intermittent synchrony

### Higher-dimensional networks with attractive coupling

Moving to more complex and physically significant examples, we consider, initially, autonomous Kuramoto networks of the form

8 $$\begin{aligned} {\dot{\theta }}_j(t) = \omega _j + \frac{K}{N}\sum _{k=1}^{N}\sin (\theta _k(t)-\theta _j(t)). \end{aligned}$$

The network size is N=100. The natural angular frequencies $\omega _j$ were randomly sampled from a Gaussian distribution of mean $\mu _\omega =\pi$ rad/s and standard deviation $\sigma _\omega =0.5$ rad/s. After this selection of natural angular frequencies, the system (8) was simulated for varying non-negative values of *K*.

The results are shown in Fig. 1 (see Fig. S2 in the Supplementary Information for more gradual *K*-increments). We see that the critical coupling strength [6] for frequency synchronisation occurs between K=1.5 and K=1.6 rad/s. The new order parameter settles at a value of 1 when *K* is greater than the critical coupling strength for frequency synchronisation. By contrast, the Kuramoto order parameter is seen to settle towards a value clearly below 1, whose closeness to 1 increases as the coupling strength is further increased. Similarly, the angular velocity order parameter remains at exactly 0 when K=0, while the Kuramoto order rapidly fluctuates at a low level. While not a transformational difference in this relatively simple case, this further illustrates the conceptual distinctions between the two parameters.

**Fig. 3:**
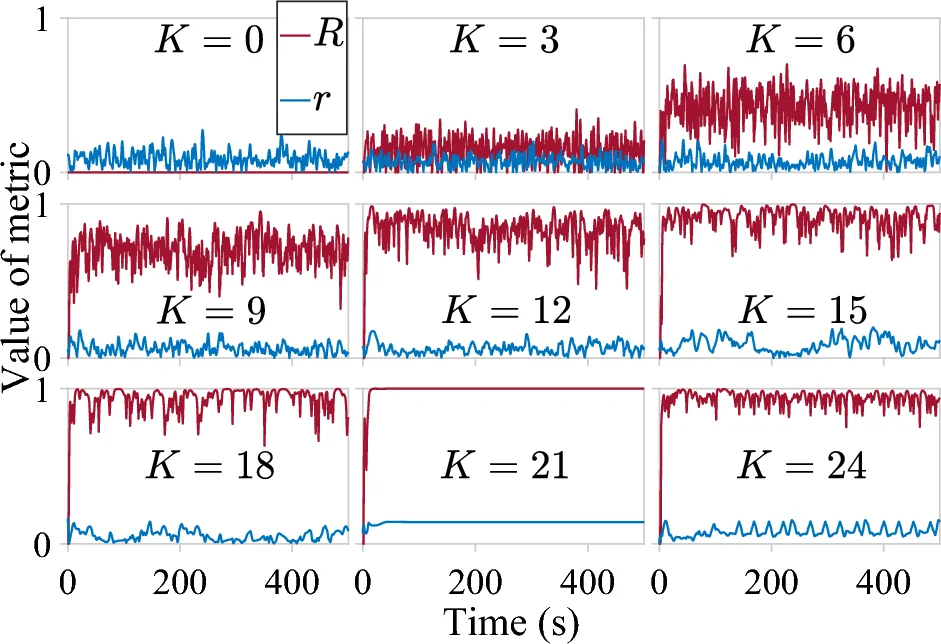
The order parameters for the autonomous Kuramoto network as specified in (10) with N=100, $\mu _\omega =\pi$ rad/s, $\sigma _\omega =0.5$ rad/s, $\alpha _{\{j,k\}} \in \{\pm 1\}$ (with even split between -1 and 1), and with coupling strength *K* having the indicated values as measured in rad/s. The value of the Kuramoto order parameter (blue line) remains at approximately the same value for all coupling strengths. In contrast, the new order parameter (red line) increases in value up to K=21 where it is equal to 1, indicating that the oscillators are phase-locked

We next consider a non-autonomous version of (8), of greater relevance to open complex systems [22],

9 $$\begin{aligned} {\dot{\theta }}_j(t) = \omega _j + M\omega _j\sin (\omega _{m}t)+ \frac{K}{N}\sum _{k=1}^{N}\sin (\theta _k(t)-\theta _j(t)), \end{aligned}$$

where the oscillators’ natural angular frequencies $\omega _j + M\omega _j\sin (\omega _{m}t)$ are time-dependent. We take the centre frequencies $\omega _j$ of the sinusoidal frequency modulation to be the same as the values of the time-independent natural frequencies $\omega _j$ in the autonomous case (8). We take M=0.5, $\omega _m=0.1$ rad/s and the coupling strength K=1.578 rad/s, which is approximately the critical coupling strength of the autonomous case (8).

The results are shown in Fig. 2, where we see *intermittent synchronisation* [32, 43, 44]. This is indicated by transitions between times when the new order parameter is approximately constant at 1 and times when the new order parameter is oscillating over a range of values. The Kuramoto order parameter more subtly evidences transitions between different behaviours by showing transitions between time-locally smoother and time-locally rougher evolution.

**Fig. 4:**
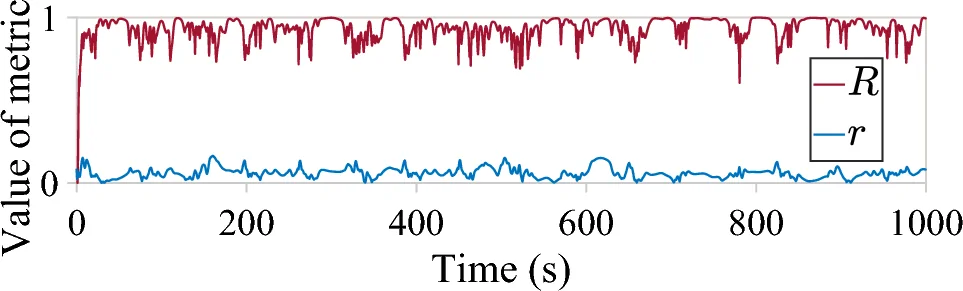
The order parameters for the non-autonomous Kuramoto network as specified in (11) with N=100, $\mu _\omega =\pi$ rad/s, $\sigma _\omega =0.5$ rad/s, M=0.5, $\omega _m=0.1$ rad/s, $\alpha _{\{j,k\}} \in \{\pm 1\}$ (with even split between -1 and 1), and K=20.503 rad/s. The Kuramoto order parameter (blue line) remains at a value close to 0, while the new order parameter (red line) reveals a series of transitions between frequency synchronisation (R=1) and non-synchronised states

### Higher-dimensional networks with symmetric mixed coupling

We next incorporate mixed, i.e. attractive and repulsive, coupling between oscillators, in an extension to yet more complex and heterogeneous physical systems such as neural networks [16, 45]. In the introduction of [33] are mentioned three forms of Kuramoto model involving mixed attractive and repulsive coupling; the first is symmetric coupling, which we consider now. We will just consider the case of networks with a uniform coupling-strength magnitude *K*. Again we start with the autonomous case, which takes the form

10 $$\begin{aligned} {\dot{\theta }}_j(t) = \omega _j + \frac{K}{N}\sum _{k=1}^{N}\alpha _{\{j,k\}}\sin (\theta _k(t)-\theta _j(t)), \end{aligned}$$

where N=100 and the value $\alpha _{\{j,k\}}=\alpha _{\{k,j\}}$ is equal to either 1 or -1 for each distinct *j*, *k*. The case j=k does not matter, since the sine term is 0 for j=k, but for this case, for the numerics we set $\alpha _{\{j,j\}}=0$. For further details see Sec. III of the Supplementary Information.

Results are shown in Fig. 3 (see also Figs. S3 and S4 in the Supplementary Information). As *K* increases, the new order parameter is clearly seen to also steadily increase. Thus, the new order parameter is able to quantify the level of coupling-induced synchrony quite clearly. By contrast, across the varying *K*, the Kuramoto order parameter remains within roughly the same band fairly close to 0. At K=21 rad/s, we appear to have phase locking, as seen by the new order parameter settling towards 1 (and evidenced indirectly by the Kuramoto order parameter settling towards a constant value). However, unlike in the case of purely attractive coupling, this phase locking does not persist as *K* is further increased; this is again seen in the new order parameter, which no longer stays at 1 for K=24. Thus, we have clearly demonstrated the strength and utility of the angular velocity order parameter, in its ability both to detect qualitative synchronisation phenomena and quantify varying degrees of synchronisation, in situations where the Kuramoto order parameter cannot do so.

A significant topic for future investigation would be to understand the dynamics in regimes of intermediate values of *R*, such as seen for K=6 rad/s. In general, intermediate *R* values would arise both in the situation that (i) few oscillators are frequency-synchronised but the spread of angular velocities is narrowed by the coupling, and (ii) an intermediate proportion of the oscillators have become frequency-synchronised.

Once again, we now consider a non-autonomous version of (10),

11 $$\begin{aligned} {\dot{\theta }}_j(t) = \omega _j + M\omega _j\sin (\omega _{m}t)+ \frac{K}{N}\sum _{k=1}^{N}\alpha _{\{j,k\}}\sin (\theta _k(t)-\theta _j(t)). \end{aligned}$$

The values of $\alpha _{\{j,k\}}$ are the same as for the autonomous case (10), and all other parameters except *K* are the same as for (9). We take K=20.503 rad/s, which is approximately equal to one of the critical values for a transition into frequency synchronisation for the autonomous system (10).

Results are shown in Fig. 4. The new order parameter indicates a fairly complex pattern of transitions into and out of time-locally near-perfect frequency synchronisation. This seems to match the high density of *K*-parameterised transitions into and out of frequency synchronisation for the autonomous case (10) (see Fig. S4 in the Supplementary Information for *K*-increments of 0.001 rad/s in the autonomous case (10)).

### Higher-dimensional networks with asymmetric mixed coupling

The second form of mixed attractive and repulsive coupling model mentioned in the introduction of [33] is highly relevant to a range of physical open systems, including analysis of swarmalators [46, 47], quantum phase oscillators [13–15] and excitatory and inhibitory neurons, the latter of which the same authors study in their subsequent paper [34]. We will again just consider the case of networks with a uniform coupling-strength magnitude *K*, giving the model

12 $$\begin{aligned} {\dot{\theta }}_j(t) = \omega _j + \frac{K}{N}\sum _{k=1}^{N}\alpha _k \sin (\theta _k(t)-\theta _j(t)), \end{aligned}$$

where for each *k*, $\alpha _k$ is equal to either 1 (for an excitatory neuron $\theta _k$) or -1 (for an inhibitory neuron $\theta _k$). The third of the three types of model mentioned in the introduction of [33] (which is the main subject of [33]) is a prototype of conformist and contrarian oscillators. Again considering just the case of networks with a uniform coupling-strength magnitude *K*, the model takes the form

13 $$\begin{aligned} {\dot{\theta }}_j(t) = \omega _j + \frac{\alpha _j K}{N}\sum _{k=1}^{N} \sin (\theta _k(t)-\theta _j(t)), \end{aligned}$$

where for each *j*, $\alpha _j$ is equal to either 1 (for a conformist oscillator $\theta _j$) or -1 (for a contrarian oscillator $\theta _j$). Due to our assumption of a uniform coupling-strength magnitude, we can transform between the form (12) and the form (13) by the following simple change of variables (which works in both directions):

14 $$\begin{aligned} \theta _j \mapsto {\left\{ \begin{array}{ll} \theta _j & \text {if } \alpha _j=1 \\ \theta _j + \pi & \text {if } \alpha _j=-1. \end{array}\right. } \end{aligned}$$

When studying the excitatory-inhibitory Kuramoto network, the paper [34] by Hong and Strogatz considers both the classical Kuramoto order parameter *r*(*t*) and a modified version that, for our system (12), reduces to

15 $$\begin{aligned} s(t) = \left| \frac{1}{N} \sum _{j=1}^N \alpha _j e^{i\theta _j(t)} \right| . \end{aligned}$$

This coincides precisely with the classical Kuramoto order parameter of (13) after applying the change of variables (14). We also note that our new order parameter *R*(*t*) is preserved under the change of variables (14). Therefore, in this section, it will only be necessary to carry out numerical simulations of the model (12) and show the results for *r*(*t*), *s*(*t*) and *R*(*t*), and this will automatically provide the same information for the model (13) as well.

We consider the system (12) with N=15, with $\alpha _k=1$ for k=1,…,6 and $\alpha _k=-1$ for k=7,…,15 (so the ratio of attractive to repulsive oscillators is 2 : 3). Due to the relatively small size of the network, we do not choose the natural angular frequencies $\omega _j$ randomly, but rather, deterministically in such a manner as to approximate a Gaussian distribution of mean $\mu _\omega =\pi$ rad/s and standard deviation $\sigma _\omega =0.5$ rad/s; for further details see Sec. IV of the Supplementary Information.

**Fig. 5:**
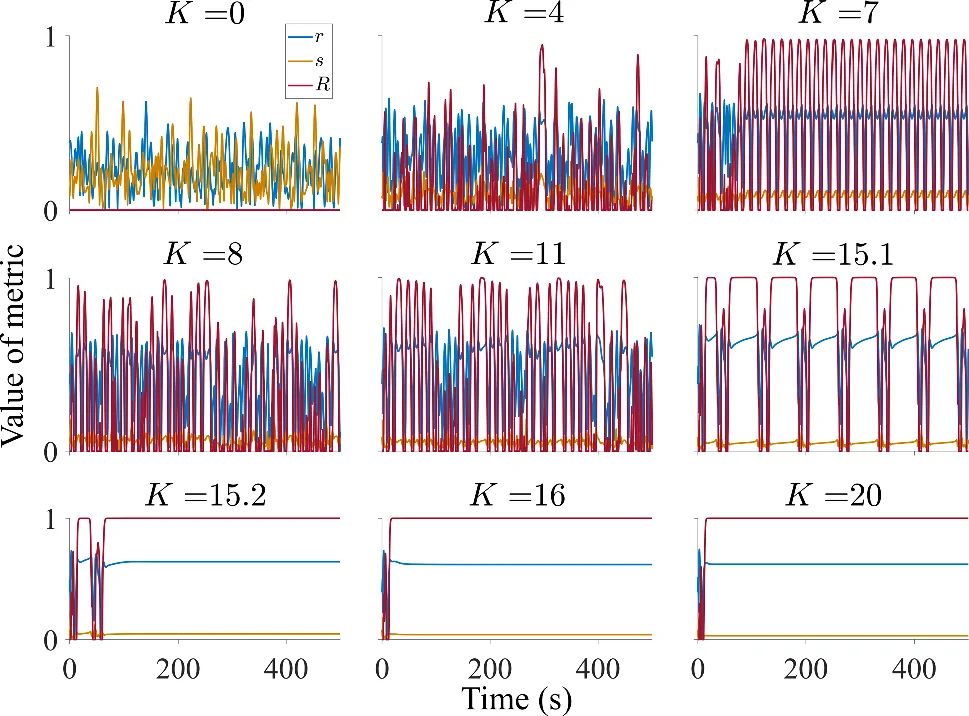
The order parameters for the autonomous Kuramoto network specified by (12) with N=15 and with $\alpha _k \in \{\pm 1\}$ in a 2 : 3 ratio of excitatory ($\alpha _k=1$) to inhibitory ($\alpha _k=-1$) oscillators. The Hong-Strogatz order parameter *s*(*t*) (yellow line, as defined by Eq. (15)) shown here for the system (12) is equivalent to the classical Kuramoto order parameter of the system (13), while the new order parameter (red line) is the same for (13) as for (12). The natural angular frequencies $\omega _j$ deterministically approximate a Gaussian distribution of mean π rad/s and standard deviation 0.5 rad/s. The coupling strength *K* has the indicated values as measured in rad/s. Above the critical coupling strength of about K=15.2 rad/s, the new order parameter takes a value of 1, indicating frequency synchronisation, while the classical Kuramoto order parameter (blue line) settles on a value between 0 and 1 and the Hong-Strogatz order parameter takes a value close to 0

Results are shown in Fig. 5 (see Fig. S5 in the Supplementary Information for more gradual *K*-increments). We see that for sufficiently large *K* (larger than a critical value of about 15.2 rad/s), *R*(*t*) is equal to 1, implying synchronisation, while the Kuramoto order parameter *r*(*t*) is not close to 1. Moreover, *s*(*t*) is close to 0, implying that for the system (13), the synchronised state is one of ‘near-perfect disorder’ from the perspective of the classical Kuramoto order parameter. The fact that *r*(*t*) is not close to 1 for the system (12) persists as *K* is increased further: we provide evidence in Sec. IV of the Supplementary Information that as $K \rightarrow \infty$, the post-transient value of *r*(*t*) tends to about 0.7 and the post-transient value of *s*(*t*) tends to 0.

Speaking from the perspective of the investigations carried out in [34], the papers [33, 34] assert that the excitatory-inhibitory form of the Kuramoto model yields nothing qualitatively new compared with attractive-coupling-only Kuramoto networks. Nevertheless, in terms of what we consider in this present paper, we do find an important difference: for attractive-only networks, in the limit of large coupling strength the synchronised state has a Kuramoto order parameter tending to 1; but in our particular example of the excitatory-inhibitory form of Kuramoto network, in the limit of large coupling strength the synchronised state has a Kuramoto order parameter not tending to 1. The use of the angular velocity order parameter may hence transform the utility of excitatory-inhibitory oscillator networks for understanding complex systems as diverse as neuronal interactions and ecology [16, 45, 48].

A natural next step would be to consider larger networks of the form (12)/(13). Challenges to achieving this are discussed in Sec. IV of the Supplementary Information.

## Macroscopic phase of phase-oscillator networks

The notion of a *macroscopic phase* of a network is another tool for describing the network’s behaviour. When the Kuramoto order parameter *r*(*t*) is close to 1, i.e. when the variance of the phases is close to 0, the mean phase ϕ(t) can serve as a quantification of the macroscopic phase. By contrast, when *r*(*t*) is much less than 1, the mean phase ϕ(t) has little meaning. Nevertheless, if the phases in the network progress synchronously over some time interval, i.e. if the pairwise phase differences within the network stay roughly constant over this time interval, then the instantaneous macroscopic state of the network can still be meaningfully described by a one-dimensional phase variable Φ(t) during this time-interval. This is true even if *r*(*t*) is close to 0 throughout the time-interval (such as in the system (10) with K=21 rad/s, or the system (13) with K>15.2 rad/s). To be precise, just as having *r*(*t*) close to 1 enables one to take ϕ(t) for the macroscopic phase of the network, so by analogy, having *R*(*t*) close to 1 enables us to take a macroscopic phase Φ(t) that progresses as

$$\begin{aligned} {\dot{\Phi }}(t) = \textrm{mean}({\dot{\theta }}_1(t),\ldots ,{\dot{\theta }}_N(t)). \end{aligned}$$

It is true that if r(t)=1, or if R(t)=1 while r(t)≠0, then ${\dot{\phi }}(t)={\dot{\Phi }}(t)$. Nevertheless, when *r* is close to 0, it is possible to have *R*(*t*) staying arbitrarily close to 1 while ϕ and Φ progress at drastically different rates.

See Sec. V of the Supplementary Information for discussion of further issues concerning Φ(t).

## Conclusions and outlook

We have seen that the new order parameter *R*(*t*) offers potentially transformative new information regarding synchronisation not provided by the Kuramoto order parameter *r*(*t*). The new order parameter is especially useful for

- showing transitions into and out of time-localised frequency synchronisation in non-autonomous networks exhibiting intermittent synchronisation, which is a defining dynamic particularly in living systems [22, 49, 50],
- showing varying levels of synchrony in mixed-coupling networks, which already attracts significant interest from a variety of physical applications [16, 45, 48].

We have also seen that a high value of the new order parameter *R*(*t*) enables a macroscopic-phase description Φ(t) of the time-evolution of the state of the network, even when the Kuramoto order parameter *r*(*t*) is small and the latter is unable to provide such a description.

In this paper, we have illustrated the effectiveness of the new order parameter for theoretical and numerical investigation of synchronisation phenomena; but we also anticipate its effective application to experimental time-series data. Since, in general, angular velocities extracted from time-series data can be sensitive to measurement noise, some smoothing may be appropriate, such as the procedure described in [51] where we can take ${\dot{\theta }}_j(t)$ to be a smoothed version of a frequency time-series obtained through ridge-extraction [52] from a time-frequency representation of the signal. In all such smoothing, an important issue is the trade-off between the filtering of noise and the capturing of the fastest timescales of genuine dynamics. For experimental data from systems where the oscillators’ internal frequencies $f_j(t)$ are varying over time, tracking these variations over time may be achievable through estimation of $f_j(t)$ via measurements of context-specific proxies for $f_j(t)$, or in cases where synchronisation is fairly weak, $f_j(t)$ may be estimated directly from the signals measuring the *N* oscillatory processes through dynamical Bayesian inference [53].

The angular velocity order parameter promises to be a transformative addition to the toolbox of oscillator-network analysis methodologies. In combination with new frameworks for non-autonomous phase descriptions [31, 32, 49], the capability of phase-based descriptions to understand the complex open systems that challenge all physical disciplines [12–14, 16–18, 20–23, 30, 33, 48, 54, 55] is hugely expanded. This is especially crucial for those scenarios in which the Kuramoto order parameter provides little to no indication of the extent of synchronisation, such as for networks with a mixture of attractive and repulsive coupling [19, 30, 33, 45, 48] as illustrated in Figs. 3–5 (and in Figs. S3 and S5 in the Supplementary Information). Examples of areas that could especially benefit from our work include, but are not limited to: brain dynamics [16–18, 21, 30, 33, 56, 57], where mixed attractive and repulsive coupling is highly relevant to the study of excitatory and inhibitory neurons; physiological oscillations [58] such as in circadian rhythms [59], cardiorespiratory coupling [60], and partial synchronisation of cellular rhythms [61]; coupled climate oscillators [62]; electric current oscillations [63, 64]; the newly developing area of synchronisation in quantum phase-oscillator networks [13–15]; and other applications as discussed in [53, 65, 66].

## Supplementary Information

Below is the link to the electronic supplementary material.Supplementary file 1 (pdf 19829 KB)

## Data Availability

The data shown in the plots and videos are available upon request from the Lancaster publications and research system Pure.

## Code availability

The codes used to produce the numerical results are available upon request from the Lancaster publications and research system Pure.
