# Supplementary material for: An order parameter for synchronisation of angular velocities
Source: Eur Phys J Spec Top. 2025 Nov 3;235(21):4475–87. doi: 10.1140/epjs/s11734-025-01984-3 (PMC13525041; doi:10.1140/epjs/s11734-025-01984-3)
Supplement: Supplementary file 1 [file 11734_2025_1984_MOESM1_ESM.pdf]

An order parameter for synchronisation of angular velocities
SUPPLEMENTARY INFORMATION

Julian Newman
*Department of Mathematics and Statistics, University of Exeter, Exeter, UK**

Joe Rowland Adams
Physics Department, Lancaster University, Lancaster, LA1 4BY
Department of Mathematics and Statistics, Lancaster University, Lancaster, LA1 4BY and
Aston Digital Futures Institute, Aston University, Birmingham, B4 7ET

Philip T. Clemson and Aneta Stefanovska
Physics Department, Lancaster University, Lancaster, LA1 4BY

I. PROOF OF VARIANCE FORMULA

Here, we prove the link between the Kuramoto order parameter of a collection of phases and the variance of the collection of phases as described in the Main Text.

Theorem. *Given values $\theta_1, \dots, \theta_N \in \mathbb{R}$ for some $N \geq 1$, let*

$$\begin{aligned} \mu &= \frac{1}{N}\sum_{j=1}^{N} e^{i\theta_j} \\ r &= |\mu| \\ \phi &= \begin{cases} \arg(\mu) & \text{if } \mu \neq 0 \\ \text{an arbitrary real number} & \text{if } \mu = 0. \end{cases} \end{aligned}$$

Then

$$1 - r = \frac{1}{2N}\sum_{j=1}^{N} \left| e^{i\theta_j} - e^{i\phi} \right|^2 .$$

In particular, if $\mu = 0$ then

$$\frac{1}{N}\sum_{j=1}^{N} \left| e^{i\theta_j} - e^{i\phi} \right|^2 = 2.$$

Lemma. *For any values $\theta, \phi \in \mathbb{R}$,*

$$\cos(\theta - \phi) = 1 - \tfrac{1}{2}\left| e^{i\theta} - e^{i\phi} \right|^2 .$$

Proof of Lemma. Letting $\delta := \theta - \phi$, we have

$$\begin{aligned} \text{RHS} &= 1 - \tfrac{1}{2}\left| (e^{i\delta} - 1)e^{i\phi} \right|^2 \\ &= 1 - \tfrac{1}{2}\left| e^{i\delta} - 1 \right|^2 \\ &= 1 - \tfrac{1}{2}\left((\cos(\delta) - 1)^2 + \sin^2(\delta) \right) \\ &= 1 - \tfrac{1}{2}\left(\cos^2(\delta) - 2\cos(\delta) + 1 + \sin^2(\delta) \right) \\ &= 1 - \tfrac{1}{2}\left(2 - 2\cos(\delta) \right) \\ &= \cos(\delta) \\ &= \text{LHS}. \end{aligned}$$

□

Proof of Theorem. By the definition of r and ϕ in terms of μ, we have

$$\mu = re^{i\phi},$$

and hence

$$\begin{aligned} r &= \mu e^{-i\phi} \\ &= \left(\frac{1}{N}\sum_{j=1}^{N} e^{i\theta_j} \right) e^{-i\phi} \\ &= \frac{1}{N}\sum_{j=1}^{N} e^{i(\theta_j - \phi)} \\ &= \operatorname{Re}\left(\frac{1}{N}\sum_{j=1}^{N} e^{i(\theta_j - \phi)} \right) \quad \text{since } r \text{ is real} \\ &= \frac{1}{N}\sum_{j=1}^{N} \cos(\theta_j - \phi) \\ &= \frac{1}{N}\sum_{j=1}^{N} \left(1 - \tfrac{1}{2}\left| e^{i\theta_j} - e^{i\phi} \right|^2 \right) \quad \text{by the Lemma} \\ &= 1 - \frac{1}{2N}\sum_{j=1}^{N} \left| e^{i\theta_j} - e^{i\phi} \right|^2 \end{aligned}$$

as required. In particular, if $\mu = 0$, then $r = 0$ and so we have

$$1 = \frac{1}{2N}\sum_{j=1}^{N} \left| e^{i\theta_j} - e^{i\phi} \right|^2 ,$$

i.e.

$$\frac{1}{N}\sum_{j=1}^{N} \left| e^{i\theta_j} - e^{i\phi} \right|^2 = 2$$

as required. □

II. RESULTS FOR TWO-OSCILLATOR SYSTEM

We consider the system (7) in the Main Text.

* j.m.i.newman@exeter.ac.uk

The phase difference $\psi(t) = \theta_2(t) - \theta_1(t)$ satisfies

$$\dot{\psi}(t) = \omega_2 - \omega_1 - K\sin(\psi(t)).$$

Consequently, if the coupling is sufficiently strong, namely if

$$|K| > \omega_2 - \omega_1, \qquad (\dagger)$$

then the system (7) of the Main Text has a stable phase-locked state. Namely,

$$\theta_2(t) - \theta_1(t) = \begin{cases} \psi_* & \text{if } K > \omega_2 - \omega_1 \\ \pi - \psi_* & \text{if } K < \omega_1 - \omega_2 \end{cases}$$

with

$$\psi_* = \arcsin(\tfrac{\omega_2-\omega_1}{K}) \in (-\tfrac{\pi}{2}, \tfrac{\pi}{2}).$$

By contrast, if

$$|K| < \omega_2 - \omega_1,$$

i.e. the coupling is not strong enough, there is no phase-locked state. Let us now consider the two order parameters:

- Under weak coupling $K \approx 0$, for every solution $(\theta_1(t), \theta_2(t))$ of (7), our new order parameter $R(t) = \max\left(1 - \left(\frac{\dot{\psi}(t)}{\omega_2-\omega_1}\right)^2, 0\right)$ will have $R(t) \approx 0$ (absence of order) at all time t, while the Kuramoto order parameter $r(t) = |\cos(\frac{\psi(t)}{2})|$ will oscillate over the full range from 0 to 1.
- Under attractive coupling fulfilling (†), for the stable phase-locked state, our new order parameter is given by $R = 1$ (perfect order) at all time, while the Kuramoto order parameter is given by $r = |\cos(\frac{\psi_*}{2})|$ at all time. As $K \to \infty$, we have $\psi_* \to 0$ and so $r \to 1$; in other words, under the Kuramoto order parameter, very strong attractive coupling gives near-perfect order.
- Under repulsive coupling fulfilling (†), for the stable phase-locked state, our new order parameter is still given by $R = 1$ (perfect order) at all time, while the Kuramoto order parameter is given by $r = |\sin(\frac{\psi_*}{2})|$ at all time. As $K \to -\infty$, we have $\psi_* \to 0$ and so $r \to 0$; in other words, under the Kuramoto order parameter, very strong repulsive coupling gives near-perfect *dis*order. This contrasts with the perfect order under our new order parameter.

We now provide numerical illustration. Numerics are performed using a Runge-Kutta 4-step numerical integration algorithm with an integration step of 0.001 s, just as in the simulations in the Main Text. We take $\omega_1 = 3$ rad/s and $\omega_2 = 3.5$ rad/s, so that the critical coupling strength for frequency synchronisation is $\omega_2 - \omega_1 = 0.5$ rad/s. Results are shown in Fig. S1. For each K-value, the two initial phases $\theta_1(0)$ and $\theta_2(0)$ were randomly selected from the uniform distribution on $[-\pi, \pi]$.

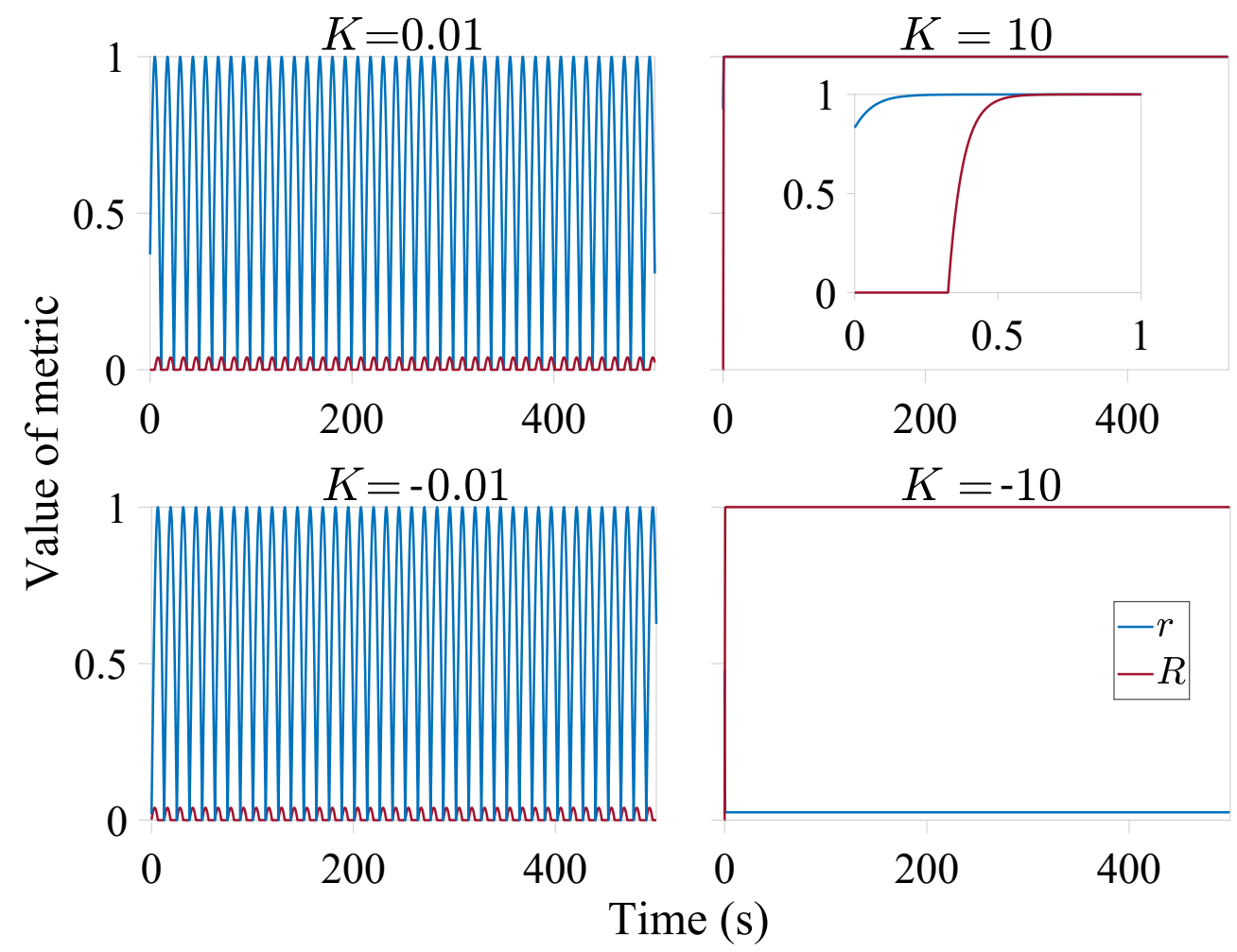

FIG. 1. Comparison of order parameters for the system (7) in the Main Text, with coupling strength K having the indicated values as measured in rad/s. Here, positive values of K correspond to attractive coupling, while negative values of K correspond to repulsive coupling.

III. NUMERICS FOR HIGHER-DIMENSIONAL SYSTEMS WITH SYMMETRIC COUPLING

For the system (10) in the Main Text, the values $\alpha_{\{j,k\}}$ with $j \neq k$ were obtained as follows. Using the MatLab `randperm` function, a random enumeration of the $\frac{N(N-1)}{2}$ different 2-element subsets $\{j,k\}$ of $\{1,\ldots,N\}$ was constructed, and then for all $\{j,k\}$ in the first half of the list, $\alpha_{\{j,k\}}$ was set to -1, while for all $\{j,k\}$ in the first half of the list, $\alpha_{\{j,k\}}$ was set to 1. The values of ω_j were taken to be the same as for the attractive coupling case equation (8) in the Main Text. The system (10) was simulated for varying non-negative values of K. For each K-value, the initial phases were randomly selected from the uniform distribution on $[-\pi, \pi]$.

In the video in Fig. S2, we show numerics for the system (8), exactly as in Fig. 1 in the Main Text but with K increasing in increments of 0.1 rad/s from 0 up to 2.6 rad/s.

In the video in Fig. S3, we show numerics for the system (10) in the Main Text, exactly as in Fig. 3 of the Main Text but with K increasing in increments of 0.5 rad/s from 0 up to 60 rad/s.

In the video in Fig. S4, we show numerics for the system (10) in the Main Text, exactly as in Fig. 3 of the Main Text but with K increasing in increments of 0.001 rad/s from 20.500 up to 20.599 rad/s. Even at this very fine K-resolution, we see multiple transitions in and out of frequency synchronisation.

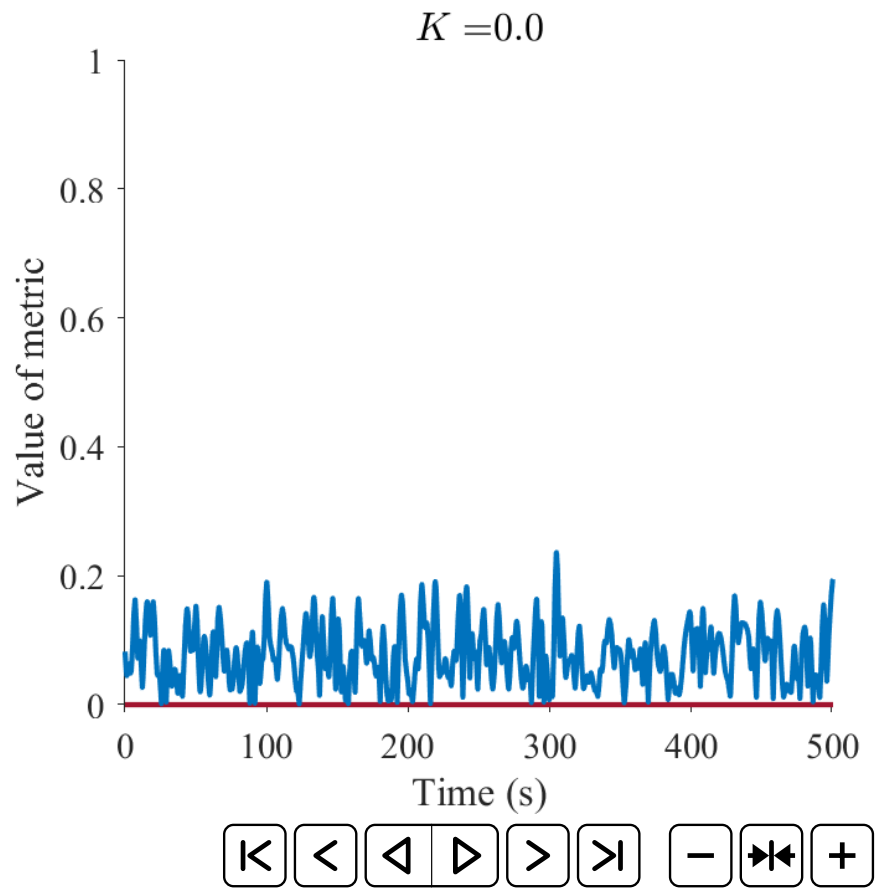

FIG. 2. An animation showing the order parameters for the system (8) with the value of K increased by 0.1 rad/s from 0 to 2.6 rad/sec in sequential frames.

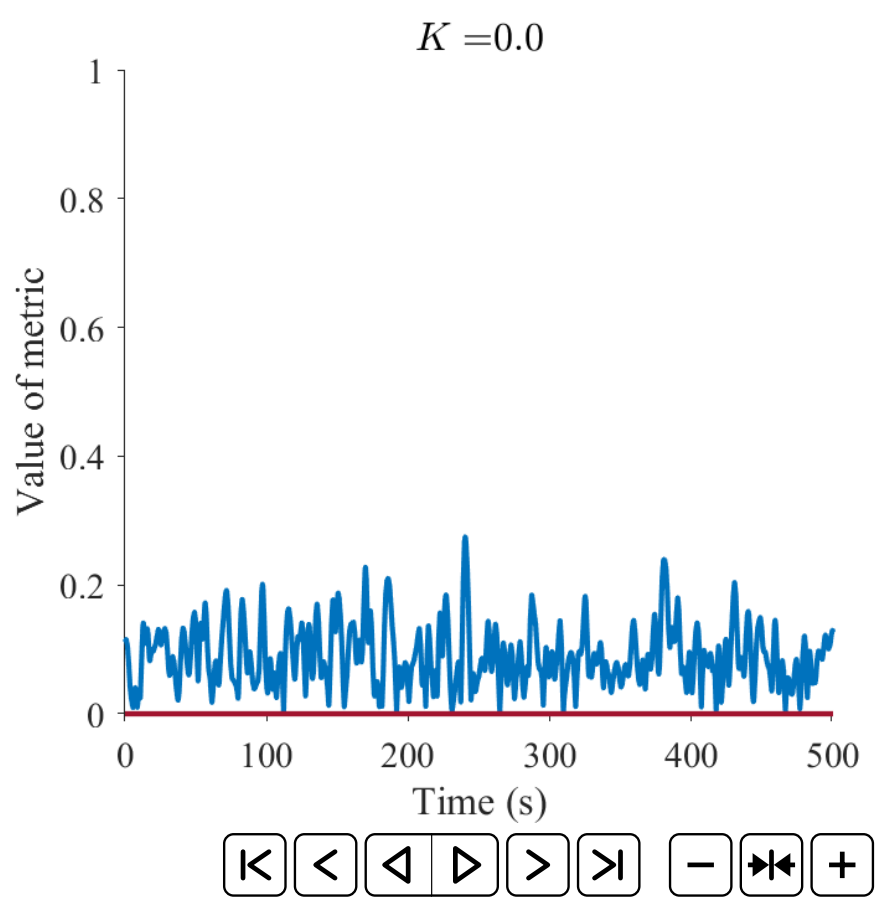

FIG. 3. An animation showing the order parameters for the system (10) with the value of K increased by 0.5 rad/s from 0 to 60 rad/s in sequential frames.

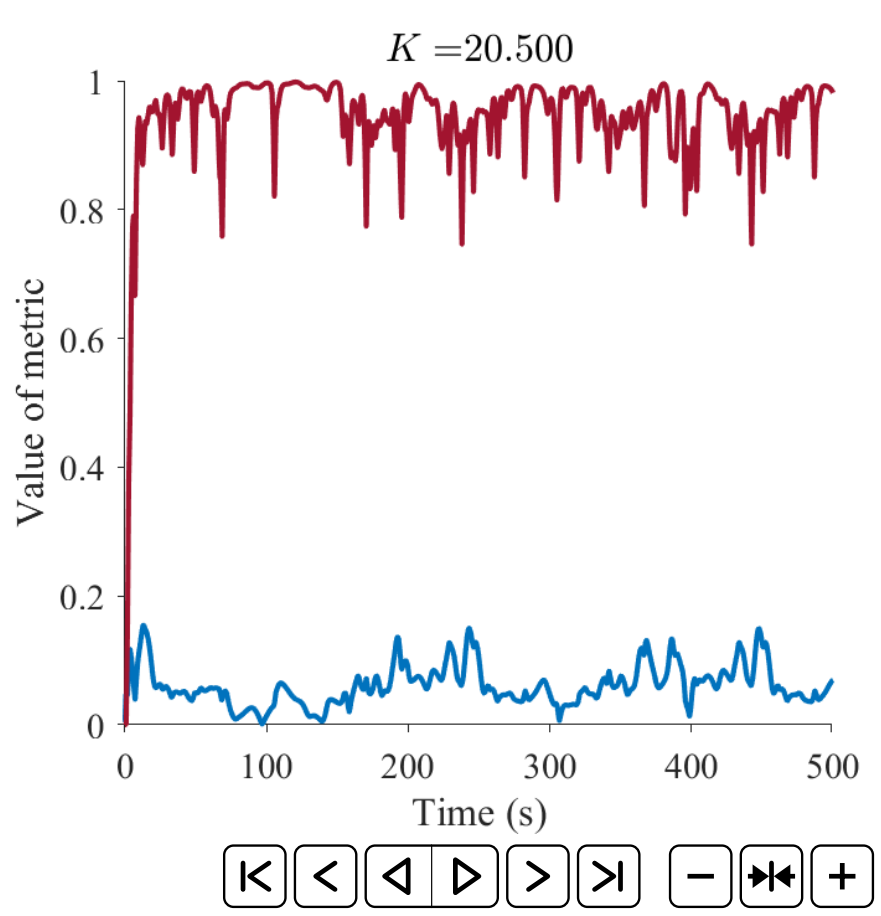

FIG. 4. An animation showing the order parameters for the system (10) with the value of K in increments of 0.001 rad/s from 20.500 to 20.599 rad/s in sequential frames.

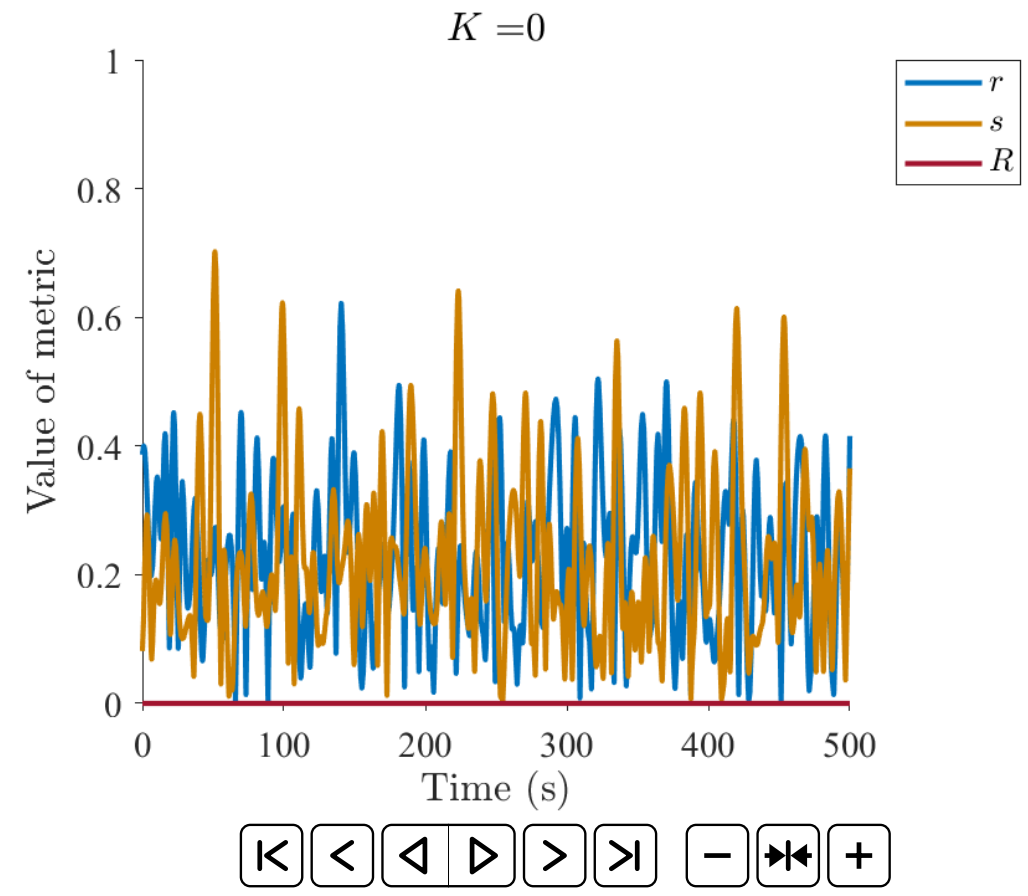

FIG. 5. An animation showing the order parameters for the system (12) with the value of K increased by 0.1 rad/s from 0 to 30 rad/sec in sequential frames.

IV. NUMERICS FOR HIGHER-DIMENSIONAL SYSTEMS WITH ASYMMETRIC COUPLING

For the system (12) in the Main Text, the values of ω_j in rad/s are given by

$$\omega_j = \begin{cases} \texttt{norminv}(\frac{j}{7}, \pi, 0.5) & j = 1, \ldots, 6 \\ \texttt{norminv}(\frac{j-6}{10}, \pi, 0.5) & j = 7, \ldots, 15 \end{cases}$$

where the MATLAB function $\texttt{norminv}(p, \mu, \sigma)$ returns the inverse of the cumulative distribution function of the normal distribution of mean μ and standard deviation σ, evaluated at the probability value p.

In the video in Fig. S5, we show numerics for the system (12), exactly as in Fig. 5 in the Main Text but with K increasing in increments of 0.1 rad/s from 0 up to 30 rad/s.

We now consider the large-K-limiting behaviour of $r(t)$ and $s(t)$ for the system (12). Setting $t_{\text{fast}} = Kt$, we can re-express Eq. (12) as

$$\frac{\mathrm{d}\theta_j}{\mathrm{d}t_{\text{fast}}} = \frac{\omega_j}{K} + \frac{1}{N}\sum_{k=1}^{N} \alpha_k \sin(\theta_k - \theta_j).$$

So then, to estimate the post-transient behaviour of (12) in the limit of large K, we can consider the system

$$\dot{\theta}_j(t) = \frac{1}{N}\sum_{k=1}^{N} \alpha_k \sin(\theta_k(t) - \theta_j(t)), \qquad (\dagger\dagger)$$

with the same values of N and α_k as in (12). Results are shown in Fig. S6. At the end of the simulation (at time $t = 500$ s), we have $r(t) \approx 0.6864$ and $s(t) \approx 1.00 \times 10^{-10}$, suggesting that for the model (12),

$$\lim_{K\to\infty} \lim_{t\to\infty} r(t) \approx 0.7$$
$$\lim_{K\to\infty} \lim_{t\to\infty} s(t) = 0.$$

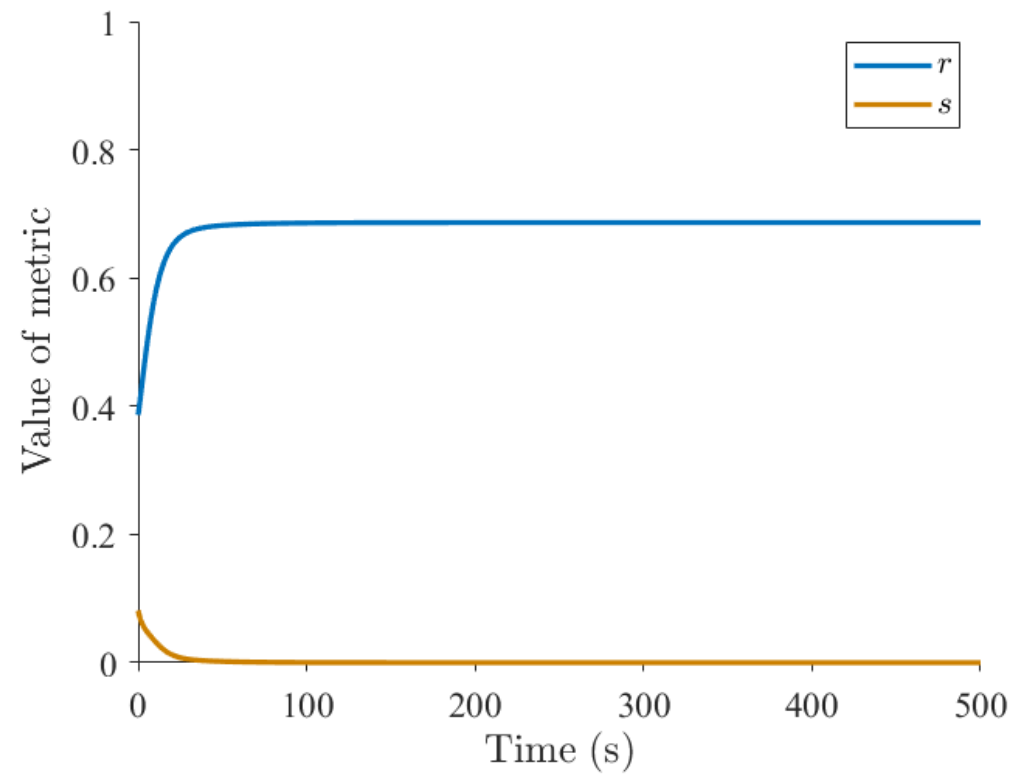

FIG. 6. Order parameters for the system (††).

In this paper, we have used a relatively small network ($N = 15$) for the system (12). Obtaining clear results for much larger N appears to be a considerably greater numerical challenge: Firstly, the coupling strengths K required to achieve synchronisation seem to be much greater for larger N, requiring a much finer integration step to ensure sufficiently accurate simulation. Secondly, for such large coupling strengths, even while the periodicities of the oscillators appear to be closely synchronised, the actual instantaneous angular velocities $\dot{\theta}_j(t)$ at each time t can have a variance that is not small compared to the variance of the unperturbed frequencies ω_j, making $R(t)$ not close to 1; and so in this case, if the variance of the angular velocities tends to 0 as $t \to \infty$, then this phenomenon of $R(t)$ failing to be close to 1 will only be transient, but it will be an extremely long transient—which, when combined with the required finer integration step, will mean that vast amounts of computation are required to observe the actual long-term behaviour of $R(t)$.

V. FURTHER DISCUSSION OF MACROSCOPIC PHASE

A noteworthy difference between the variable Φ and the mean phase ϕ is that the choice of which collective state $(\theta_1, \dots, \theta_N)$ to calibrate as $\Phi = 0$ is not tied to a pre-determined choice of which states of the individual oscillators θ_j to calibrate as $\theta_j = 0$. Rather, just as the choice of $\theta_j = 0$ is arbitrary for the individual oscillators, so likewise the choice of which collective state $(\theta_1, \dots, \theta_N)$ to calibrate as $\Phi = 0$ is arbitrary. This is because, as we described at the start of the Main Text, the concept of synchronisation of angular velocities does not make reference to any identification of phase-calibrations between different oscillators. The point is that the *change* in $\Phi(t)$ over time t matches the synchronised change in phase of the individual oscillators in the network.

We warn that $\Phi(t)$ cannot be taken as a self-consistent description of the state of the network over time-intervals sufficiently long to allow for a significant proportion of the pairwise phase differences in the network to undergo a significant level of drift, even if $R(t)$ is close to 1 at all times during this a time-interval. This is because there exist continuous paths in the $(\theta_1, \dots, \theta_N)$-state space (the N-torus) that start and end at the same point, such that tracing the evolution of Φ continuously over the path will give a final value of Φ that differs from the initial value by a non-zero integer multiple of $\frac{2\pi}{N}$. Still, if over a given time-interval, the proportion of pairs of oscillators undergoing significant drift in phase difference is sufficiently small, $\Phi(t)$ may provide a suitable *approximate* description of macroscopic phase over this time-interval.
